# Investigation of tryptophan to kynurenine degradation in response to interferon-γ in melanoma cell lines

**DOI:** 10.1016/j.bbrep.2023.101612

**Published:** 2023-12-19

**Authors:** Helena Tassidis, Skaidre Jankovskaja, Kassem Awad, Lars Ohlsson, Anette Gjörloff Wingren, Anna Gustafsson

**Affiliations:** a Department of Natural Science, Kristianstad University, Kristianstad, Sweden; b Department of Biomedical Science, Faculty of Health and Society, Malmö University, Malmö, Sweden; c Biofilms – Research Center for Biointerfaces, Malmö University, Malmö, Sweden

**Keywords:** IDO-1, Interferon-γ, Kynurenine, Melanocytes, melanoma, Programmed death ligand 1, Tryptophan

## Abstract

**Background and aim:**

Melanoma is a fatal form of skin cancer that carries a grave prognosis if the cancer cells spread and form metastases. The Kynurenine (Kyn) pathway is activated by the enzyme indoleamine 2,3-dioxygenase 1 (IDO-1) and has been shown to have a role in tumour progression. We have previously shown that interferon-γ (IFN-γ) acts as an inducer of tryptophan (Trp) degradation to Kyn in keratinocytes of the basal layer in a 3D epidermis model. Before extending our reconstructed human epidermis model to not only contain keratinocytes but also fibroblasts and melanocytes/melanoma cells, we have in this study set out to investigate possible differences between primary adult melanocytes and six melanoma cell lines regarding the expression of the immune checkpoint inhibitors IDO-1 and programmed death ligand 1 (PD-L1) together with Kyn production.

**Methods:**

The melanocytes and melanoma cells were stimulated with 1–20 ng/ml of IFN-γ and the levels of Trp to Kyn degradation were monitored with high-performance liquid chromatography (HPLC). To analyze the viability of the cell types after IFN-γ treatment, an MTT assay was performed. mRNA quantity of IDO-1, PD-L1 and IFN-γ receptor (IFN-GR1) was analyzed with qPCR.

**Results:**

After 24 h, only the metastatic cell line WM-266-4 was affected by all concentrations of IFN-γ, whereas at 48 h, the higher IFN-γ concentrations gave a more pronounced effect on the viability in all cell types. Trp was detected at various levels in the culture medium from all cell types before and after IFN-γ treatment. The degradation to Kyn was detected in primary melanocytes, Mel Juso, and Mel Ho cell lines after 24 h of treatment and low levels of IFN-γ. However, the higher concentration of IFN-γ, 20 ng/ml, induced Kyn to various degrees in all cell types after 24 h. The change in mRNA quantity of IDO-1 and PD-L1 was similar in all cell types.

**Conclusion:**

To conclude, no significant difference in upregulation of the immune checkpoint inhibitors PD-L1 and IDO-1 was seen between primary tumour and metastatic melanoma. IFN-γ stimulation of melanocytes and different stages of melanoma cell lines resulted in an increased Kyn/Trp ratio in the more aggressive melanoma cells when a high concentration was used (20 ng/ml) but when a lower concentration of IFN-γ (5 ng/ml) was used an increased Kyn/Trp ratio were detected in media from primary melanocytes and early-stage melanoma.

## Highlights

• IFN-γ stimulation of melanocytes and different stages of melanoma cell lines results in an increased Kyn/Trp ratio.
• IFN-γ raises Kyn/Trp ratio more in aggressive melanoma than early-stage melanoma.
• No significant IFN-γ-induced differences in PD-L1 and IDO-1 expression across stages.

## 1 Introduction

Melanoma is an aggressive skin cancer and can be cured if diagnosed at an early stage. The clinical examination should always be confirmed with dermatoscopy [1]. Sequential digital dermatoscopy and full body photography can be used in high-risk patients to improve the detection of early melanoma. However, a significant number of patients will not have a favourable survival rate, since melanoma often develops into a metastatic disease [2]. Even though recent advances in therapies for metastatic melanoma have improved, late-stage melanoma has a poor prognosis with a 5-year survival rate of 15–20 % [3,4]. Recent advancements in our understanding of immunotherapies and combination therapies have revolutionized the management of advanced melanoma. Immune checkpoint molecules, including programmed cell death-1 (PD-1), programmed death ligand 1 (PD-L1), and indoleamine 2,3-dioxygenase (IDO) maintain immune tolerance under normal conditions by controlling the initiation, duration, and magnitude of immune responses [5,6]. However, during the development of tumors, these signalling pathways may inhibit the host's anti-tumour immunity, leading to a tumor immune escape [7].

Interferon-γ (IFN-γ) is a key cytokine produced by activated T cells and NK cells in the tumour microenvironment, playing an important role in coordinating the anti-tumor response [8]. The intracellular enzyme IDO-1 is expressed in dendritic cells, macrophages, and endothelial cells in the tumour microenvironment [9]. IDO-1 exhibits immune-suppressive activity through catalyzing the rate-limiting step in the degradation of l-tryptophan (Trp) into l-kynurenine (Kyn), which in turn facilitates immune escape by depleting Trp. Interestingly, IDO-1 has been shown to be expressed in tumors from breast, esophageal carcinoma, colorectal cancer, cervical squamous cell carcinoma, melanoma, and pancreatic cancer, and is upregulated by IFN-γ [9]. IDO-1 expression in primary human melanoma has been shown to significantly correlate with Breslow index describing the thickness of the melanoma, the presence of tumour infiltrating lymphocytes, and the intensity of the inflammatory infiltrate [10]. Chevolet et al. also report an independent correlation between peritumoural IDO-1 expression in primary, sentinel, and metastatic tissues of patients with melanoma [11]. In addition, decreased serum Trp concentrations have been shown to be a predictive marker in melanoma patients [12]. Kyn is toxic to lymphocytes and induces differentiation of regulatory T lymphocytes via the binding to the transcription factor aryl hydrocarbon receptor (AhR). Activation of AhR is one of the reasons why Kyn is perceived as an oncometabolite, since AhR regulates growth promoting genes in cancer cells [13].

The cell surface receptor PD-1 is expressed on various immune cells, including T- and B-lymphocytes, dendritic cells, monocytes, and natural killer cells. PD-1 has two ligands, PD-L1 and PD-L2. PD-L1 is commonly expressed on tumour cells, including melanoma, and is upregulated upon induction by cytokines such as IFN-γ [14]. Immunotherapies, including immune checkpoint inhibitors (such as anti-PD-1 and anti-IDO1 antibodies [15] and adoptive cell therapy, aim to activate and enhance the body's immune response against melanoma cells. PD-L2 is mainly expressed on activated macrophages and on dendritic cells [16]. The interaction of PD-1 on cytotoxic T lymphocytes and PD-L1 on tumour cells results in diminished T cell killing [17]. PD-L1 levels are used for prediction of therapeutic response, but variable levels occur with variations in response to tumour-targeting immune cells releasing IFN-γ.

Melanoma is a heterogeneous disease [18], which makes it more challenging in designing therapies and finding specific biomarkers. Therefore, it is important to investigate biomarkers in the early stages of the disease. Jankovskaja et al. have shown that Kyn and Trp can be sampled from the skin surface of healthy subjects, which implies that the Kyn/Trp ratio could be a promising non-invasively detectable biomarker for skin diseases [19]. We have previously established an *in vitro* human epidermis 3D model and showed that IFN-γ induces Trp degradation to Kyn in the basal layer of keratinocytes [20]. To extend this model we have investigated cell viability, mRNA levels of IDO-1, PD-L1, and the Kyn/Trp ratios in six different human melanoma cell lines as well as in primary adult melanocytes after exposure to IFN-γ. The aim of the study was to investigate the Kyn/Trp ratio in different melanomas.

## 2 Material and methods

### 2.1 Cell lines, culture media, and chemicals

The cell lines Mel Ho (ACC62), CHL-1 (CRL-9446), HT144 (HTB-63), SK-MEL-3 (HTB-69), and WM-266-4 (CRL-1676) were all purchased from ATCC/LGC Standards. Cell line Mel Juso (ACC74) was from DSMZ-German Collection of Microorganisms and Cell Cultures. Cell lines Mel Juso, Mel Ho, and CHL-1 were derived from primary cutaneous melanomas. HT144, SK-MEL-3, and WM-266-4 were derived from metastases of malignant melanomas. Normal human primary epidermal melanocytes from adult tissue (HEMa), dermal cell basal media, and adult melanocyte growth kit components were purchased from ATCC/LGC Standards. The adult melanocyte growth kit provides recombinant human insulin (5 μg/ml), ascorbic acid (50 μg/ml), l-glutamine (6 mM), epinephrine (1.0 μM), calcium chloride (1.5 mM), peptide growth factor (0.2 % v/v), and M8 supplement (1 % v/v). Dulbecco's Modified Eagle's Medium (DMEM), Minimum Essential Medium (MEM), McCoy's 5a Medium Modified, fetal bovine serum (FBS), Accutase, penicillin and streptomycin were purchased from Thermo Fischer Scientific. IFN-γ were purchased from R&D Systems. The cell viability assay 3-(4,5-Dimethylthiazol-2-yl)-2,5-diphenyltetrazolium bromide (MTT) was from Roche. RNeasy Mini Kit was purchased from Qiagen and SensiFast cDNA synthesis kit was from Bioline. SYBR Green from Thermo Fisher Scientific. The quantification of Trp and Kyn was done by using a HPLC system comprising a G1312A model binary pump, a G1322A in-line degasser, a G1316A column oven, a G1313A autosampler, and a G1315 diode array detector (Agilent 1100 Series). Chromatographic separation of Trp and Kyn was performed on a Kromasil C18 column (250 × 4.6 mm, 5 μm, 100 Å) (AkzoNobel, ES industries). NaH_2_PO_4_ from Merck. 100 % HPLC grade methanol from VWR international. Trp and Kyn (Sigma-Aldrich). Filters with molecular weight cut-off (MWCO) of 3k Da (PES modified, VWR international).

### 2.2 Culture of melanoma cells and primary melanocytes

Mel Juso and Mel Ho were cultured in DMEM with 1 g/L glucose supplemented with 10 % FBS, penicillin (400 U/ml), and streptomycin (50 μg/ml). WM-266-4 and CHL-1 were cultured in MEM supplemented with 10 % FBS, penicillin (400 U/ml), and streptomycin (50 μg/ml). HT144 and SK-MEL-3 were cultured in McCoy's 5a Medium Modified supplemented with 10 % FBS, penicillin (400 U/ml), and streptomycin (50 μg/ml). HEMa were grown in dermal cell basal media supplemented with adult melanocyte growth kit components. All cells were incubated in a humidified atmosphere with 5 % CO_2_ at 37 °C and split at 80–90 % confluency using Accutase cell detachment solution. All stimulations of melanoma cells were carried out in a culture medium supplemented with 5 % instead of 10 % FBS. The melanocytes were cultured in dermal cell basal media as described above during stimulation.

### 2.3 Cell viability test

For the cell viability test, the cells were seeded in 96-well culture plates at a density of 1 × 10^4^ cells/well. Cells were allowed to attach for 24 h and then the culture medium was exchanged for a new medium with or without IFN-γ (1.0, 5.0, or 20 ng/ml) and the cultivation was continued for 24 or 48 h. Then, 0.5 mg/ml MTT was added to the IFN-γ treated cells. After 4 h of incubation at 37 °C in 5 % CO_2_, a solubilization solution was added and the cells were incubated overnight at 37 °C in 5 % CO_2_. The optical density (OD) was measured at 550 nm with a reference wavelength of 660 nm.

### 2.4 RNA isolation, cDNA synthesis, and qPCR

For quantification of mRNA, 1 × 10^5^ cells/well were seeded in 12-well cell culture plates. Following a 24-h incubation period, the culture medium was replaced with fresh medium, either with or without IFN-γ, administered at varying concentrations (5 or 20 ng/ml). Following an additional 24-h incubation, the cells were lysed and subjected to total RNA extraction and cDNA synthesis, following the manufacturer's instructions. PCR was then carried out using SYBR Green technology. Each 20 μl sample contained 5 μl of a template cDNA, 1 μl of each primer (10 μM), 10 μl SYBR Green and 3 μl of nuclease-free water. Each reaction was run in triplicate on an LC480 LightCycler from Roche using the following program: Initial denaturation at 95 °C for 10 min, followed by 45 cycles of 95 °C during 10 s for melting, 65 °C for 10 s annealing and 72 °C for 11 s extension. The program ended with a continuous melting curve analysis measuring fluorescence from 60 to 97 °C. Relative gene levels were calculated using the ΔΔ-CT-method, using glyceraldehyde 3-phosphate dehydrogenase (GAPDH) as the reference gene. Primers were as follows:

IDO-1: fwd: GAAAGGCAACCCCCAGCTAT. rev: GGAGGAACTGAGCAGCATGT.

IFN-GR1: fwd: CATCACGTCATACCAGCCATTT. rev: CTGGATTGTCTTCGGTATGCAT.

PD-L1: fwd: TCTGGCACATCCTCCAAATG. rev: CAGTGCTACACCAAGGCATAATAAG.

GAPDH: fwd: AACAGCGACACCCACTCCTC. rev: GGAGGGGAGATTCAGTGTGGT.

### 2.5 Quantification of kynurenine and tryptophan with HPLC

For quantification of Trp and Kyn, 1 × 10^5^ cells/well were seeded in 12-well cell culture plates. After 24 h, the culture medium was exchanged for a new medium supplemented with or without IFN-γ in different concentrations (5 or 20 ng/ml). After an additional 24 h, the culture medium was collected, and aliquots were immediately frozen at −80 °C. The quantification of Trp and Kyn was done by using a HPLC system. Solvent A, 10 mM NaH_2_PO_4_ (pH 2.8) and solvent B, 100 % HPLC grade methanol were used to create a 17 min gradient to elute the analytes. The separation profile was as follows: 25 % B held for 7 min, increased to 95 % B in 4 min, and held for 4 min; then, solvent B was decreased to 25 % B in 0.1 min and held for 1.9 min. The flow rate was set to 0.9 ml/min and the column oven temperature was kept at 40 °C. Trp was monitored at 280 nm and Kyn at 360 nm.

Stock solutions of 20 mM of both Trp and Kyn were prepared in deionised water. Standards for the calibration curves (1.56–200 μM) were prepared by diluting stock solutions with appropriate the cell culture medium. Prior to HPLC analysis, in order to get rid of the proteins, all samples were pipetted into centrifugal filters with MWCO of 3k Da and centrifuged for 15 min at 12 000×*g*, at 10 °C. Cell culture samples and calibration standards were run in the same analytical batch, in triplicates. The concentration of analytes was calculated by automatic peak areas integration by OpenLAB software (Agilent, Lab Advisor Basic Software), followed by manual inspection of the data. The limit of detection (LOD) was calculated to be 0.4–0.7 μM for Kyn, and 0.3–1.1 μM for Trp. The limit of quantification (LOQ) was determined to be 1.3–2.0 μM for Kyn, and 0.9–3.3 μM for Trp. The LODs and LOQs for different cell lines were slightly different due to the difference of the cell culture medium used. The LOD and LOQ were calculated from standard calibration curves as follows; LOD = 3.3σ/slope and LOQ as LOQ = 10σ/slope, where σ is the standard error of the y-intercept from the regression analysis of the calibration standards. The LODs and LOQs calculated for each cell line can be found in Supporting *Information*, Table S1.

### 2.6 Statistics

The results, presented as mean ± standard deviation (SD), underwent thorough analysis through a two-way ANOVA followed by the Tukey test, utilizing GraphPad Prism software (version 10.1.0, GraphPad Inc., San Diego, CA, USA). To assess cell viability (MTT), comparisons were made between untreated and treated groups. Furthermore, for both mRNA quantity and kynurenine/tryptophan concentration, statistical significance was determined by contrasting the untreated group (control) with both the 5 ng/ml and 20 ng/ml treatment groups. Additionally, a direct comparison between the 5 ng/ml and 20 ng/ml treatment groups was conducted. Statistically significant differences were identified when the p-value was less than 0.05 (P < 0.05); denoted as *P ≤ 0.05, **P ≤ 0.01, ***P ≤ 0.001.

## 3 Results and discussion

**Fig. 1:**
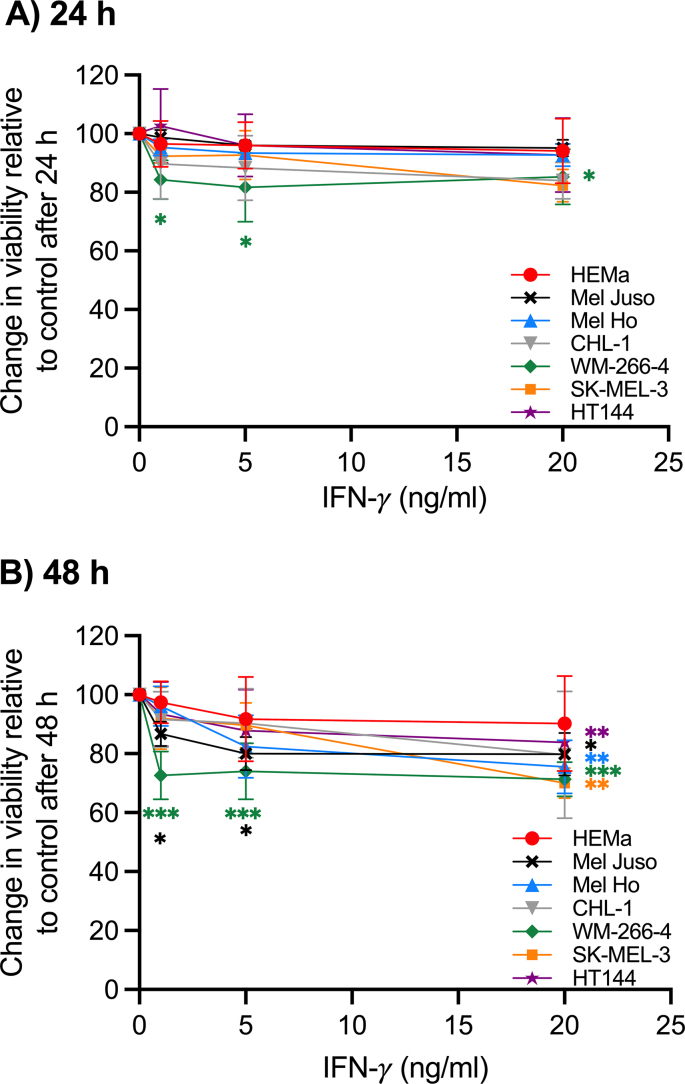
MTT-based viability of six melanoma cell lines and adult human epidermal melanocytes (HEMa) after 24 and 48 h treatment with IFN-γ in indicated concentrations. Data represent the means ± SD of triplicate determinations from three separate experiments. *P ≤ 0.05 **P ≤ 0.01 ***P ≤ 0.001 vs untreated cells.

**Fig. 2:**
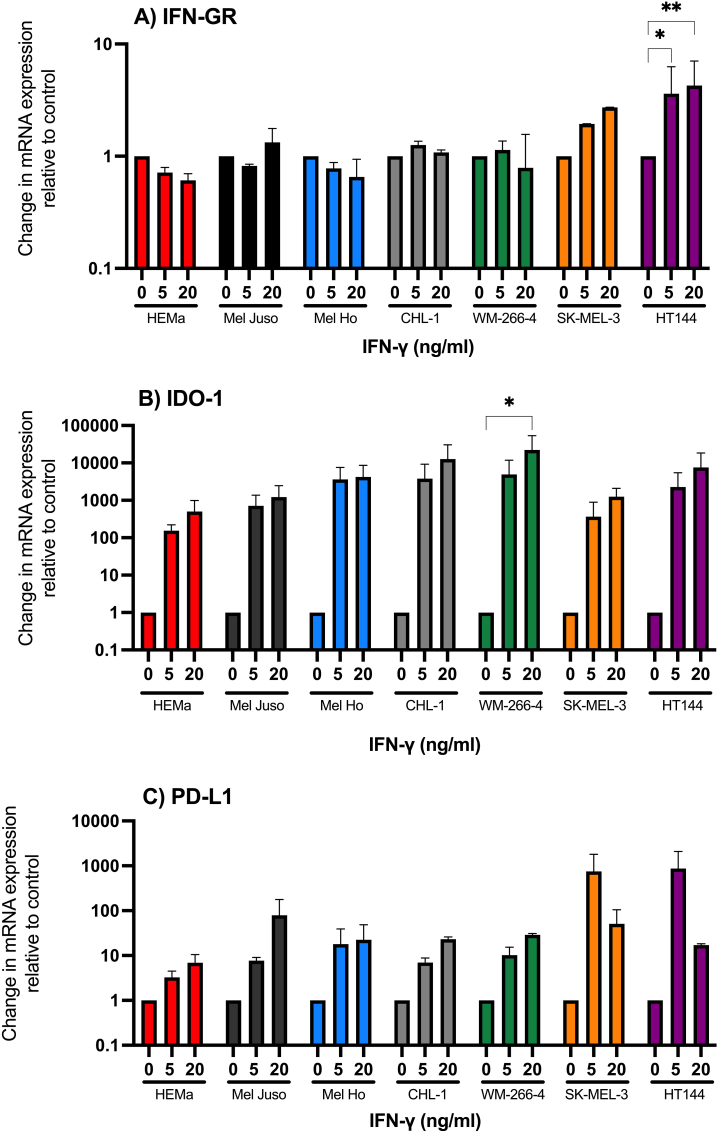
Change in mRNA quantity of IFN-GR1 (A), IDO-1 (B) and PD-L1 (C) in adult human epidermal melanocytes (HEMa) and six human melanoma cell lines (Mel Juso, Mel Ho, WM-266-4, CHL-1, SK-MEL-3, HT144) after 24 h IFN-γ treatment (5 and 20 ng/ml). The baseline expression from unstimulated cells is represented as '1′ in the figure. Data represent the means ± SD of triplicate determinations from two separate experiments. *P ≤ 0.05 **P ≤ 0.01.

Melanoma accounts for the majority of skin cancer-related deaths, making its early detection and appropriate management crucial for improved patient outcomes. The diagnosis of melanoma is primarily based on clinical examination and histopathological evaluation. Further research is needed to validate soluble markers for melanoma and their optimal use in clinical practice. We have analyzed normal melanocytes from human adult epidermal tissue, HEMa, together with cell lines of primary cutaneous melanomas (Mel Juso, Mel Ho, and CHL-1) and metastases of malignant melanomas (HT144, SK-MEL-3, and WM-266-4). To simulate the tumour microenvironment, all cell types in this study were treated for up to 48 h with 1–20 ng/ml of IFN-γ. After 24 h, only the WM-266-4 melanoma cell line was affected by all the IFN-γ concentrations, whereas at 48 h, the higher IFN-γ concentrations resulted in a significant decrease in the viability of all cells, except the primary melanocytes (HEMa) and the CHL-1 melanoma cell line (Fig. 1). Hence, in the following experiments, only 24 h stimulation was performed. Krasagakis et al. also reported that IFN-γ treatment of melanocytes did not affect cell growth [1]. Another study showed different responses of melanoma cell lines to IFN-γ treatment, where one cell line showed pronounced growth inhibition, whereas two other cell lines showed a moderate response, and one was relatively resistant to IFN-γ [21]. According to Kortylewski et al. [21], the G_0_/G_1_ arrest does not correlate with inhibition of cell growth in the four melanoma cell lines included in their study. Moreover, their study showed only a marginal increase in apoptosis [21]. This implies that IFN-γ could have a diverse effect regarding anti-proliferative properties in melanoma cell lines. The receptor for IFN-γ (IFN-GR1) was expressed in all cell types tested and the mRNA quantity was only significantly upregulated in the metastatic cell line HT144 and to a minor extent in SK-MEL-3 cells after treatment with IFN-γ. In the other cell types, no upregulation of mRNA for IFN-GR1 could be detected after stimulation with 5 or 20 ng/ml of IFN-γ (Fig. 2A).

**Fig. 3:**
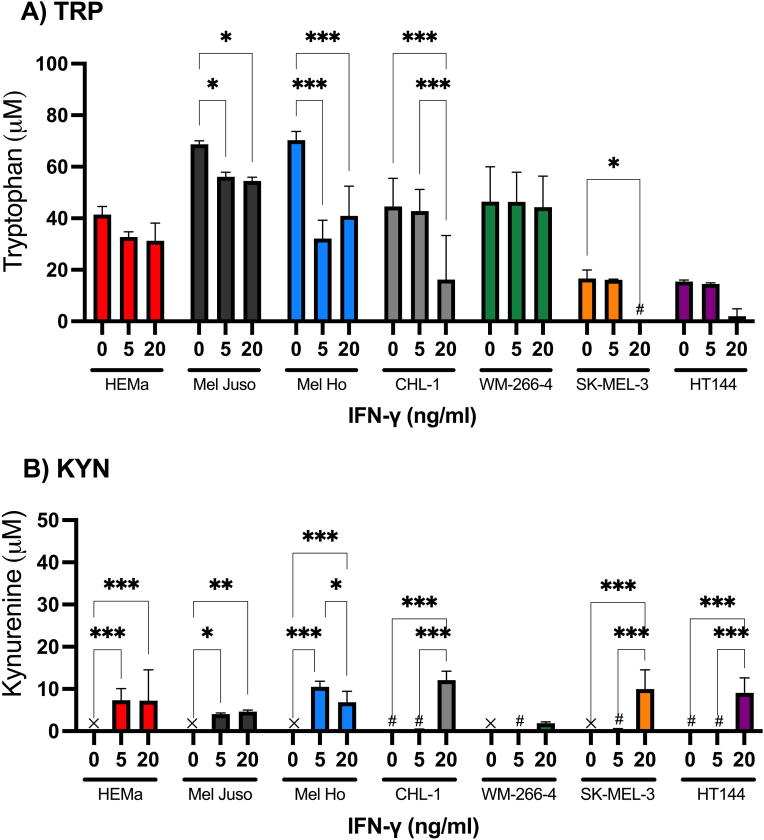
Extracellular concentrations of tryptophan (**A**) and kynurenine (**B**) in the culture medium of adult human epidermal melanocytes (HEMa) and six human melanoma cell lines (Mel Juso, Mel Ho, CHL-1, WM-266-4, SK-MEL-3, HT144) under basal conditions (0) and after 24 h IFN-γ treatment (5 and 20 ng/ml). Data represent the means ± SD of duplicate determinations from two separate experiment. # = detected but below the limit of quantification. x = not detected. *P ≤ 0.05 **P ≤ 0.01 ***P ≤ 0.001. (**C**) represents the Ratio of Kyn/Trp (means ± SD) in culture medium from stimulated cell cultures. Significance levels are denoted as follows: *P ≤ 0.05, ***P ≤ 0.001, indicating a significant difference between stimulation with 5 and 20 ng/ml IFN-γ.

Trp was detected at various levels in the culture media from all cell types before and after IFN-γ treatment (Fig. 3A). The variability in basal levels of Trp (media from unstimulated cells; ctrl) is due to the different Trp concentrations in various culture media. Statistically significant higher levels of Kyn were detected in media from primary melanocytes and in the early-stage melanoma cell lines Mel Juso, and Mel Ho after 24 h of treatment with 5 ng/ml of IFN-γ (Fig. 3B). The higher concentration of IFN-γ, 20 ng/ml, induced Kyn to various degrees in all cell types after 24 h (Fig. 3B). Interestingly, when calculating the Kyn/Trp ratios, a five to ten-fold increase for the two metastatic cell lines SK-MEL-3 and HT144 could be demonstrated after stimulation with IFN-γ 20 ng/ml, but not for the metastatic cell line WM-266-4 in this study (Fig. 3C). Kyn was not detected without IFN-γ stimulation in any cell type. IFN-γ signalling activates Jak1/Jak2-mediated STAT1 activation [17]. Importantly, one of the activated genes is interferon regulatory factor 1 (IRF1) which acts as a transcription factor when ligating to promoters such as the PD-L1 promoter. Other signalling pathways could be included in future studies, including the Erk/MAPK signalling pathway are involved [22].

Over the last decade, several new targeted therapies and immunotherapies for metastatic melanoma have been developed and shown to improve the prognosis. IFN-γ treatment upregulated mRNA quantity of the immune checkpoints IDO-1 and PD-L1 to various degrees in the different cell lines (Fig. 2B and C). No significant difference between melanocytes, early-stage (Mel Ho, Mel Jusu, and CHL-1), and late-stage melanoma cells (WM-266-4, SK-MEL-3, and HT-144) in the upregulation of IDO-1 and PD-L1 could be detected. Also, no significant correlation between the Kyn/Trp ratio and IDO-1 was found.

It has previously been shown that IFN-γ can induce the expression of PD-L1 in melanoma cell lines [23]. Atefi et al. analyzed PD-L1 expression in a panel of 51 melanoma cell lines, the expression could not be correlated to any mutation (BRAF, RAF or BRAF/RAF) [24]. The Kyn/Trp ratio in blood from stage IV melanoma patients has been shown to be increased compared to stage I-III and healthy controls [25]. At the same time, no correlation between the Kyn/Trp ratio and IDO-1 expression in circulating immune cells of the peripheral blood samples of the patients was seen. *Ex vivo* stimulation of patients' PBMCs with IFN-γ showed upregulation of IDO-1 in monocytes but not in lymphocytes, and this induction was variable between the patients. Patients with high induction of IDO-1 in monocytes had better outcomes than patients with less upregulation.

## 4 Conclusions

Here we have demonstrated that IFN-γ stimulation of melanocytes and different stages of melanoma cell lines results in a Kyn/Trp ratio that is increased in the more aggressive melanoma cells when a high concentration is used (20 ng/ml). However, with low levels of IFN-γ (5 ng/ml) increased Kyn/Trp ratios were detected in media from primary melanocytes and early-stage melanoma but not in the aggressive melanomas. Moreover, IFN-γ induction of the immune checkpoint inhibitors PD-L1 and IDO-1 was found but no significant difference between melanocytes, early-stage, and late-stage melanoma cells could be detected.

## 5 Ethics statement

All melanoma cell lines, and primary melanocytes used in this project are sourced from reputable suppliers, including ATCC/LGC Standards and DSMZ-German Collection of Microorganisms and Cell Cultures. These products are intended for research use only, and we strictly adhere to the terms and conditions specified by the suppliers. Unauthorized uses, including commercial, diagnostic, therapeutic, or any application to humans or animals, are strictly prohibited. Furthermore, prior to sample donation, written informed consent is obtained from all donors, ensuring ethical compliance in our research.

## Funding

This work was supported by the 10.13039/100003077Knowledge Foundation (grant number 20190010), and Biofilms Research Center for Biointerfaces Malmö University, Sweden.

## CRediT authorship contribution statement

**Helena Tassidis:** Writing – review & editing, Writing – original draft, Methodology, Investigation, Formal analysis, Conceptualization. **Skaidre Jankovskaja:** Methodology, Investigation, Formal analysis. **Kassem Awad:** Investigation, Formal analysis. **Lars Ohlsson:** Writing – review & editing, Investigation. **Anette Gjörloff Wingren:** Writing – review & editing, Writing – original draft, Conceptualization. **Anna Gustafsson:** Writing – review & editing, Writing – original draft, Visualization, Methodology, Investigation, Funding acquisition, Formal analysis, Conceptualization.

## Declaration of competing interest

The authors declare that they have no known competing financial interests or personal relationships that could have appeared to influence the work reported in this paper.

## Data Availability

Data will be made available on request.
