# Supplementary material for: Investigation of tryptophan to kynurenine degradation in response to interferon-γ in melanoma cell lines
Source: Biochem Biophys Rep. 2023 Dec 19;37:101612. doi: 10.1016/j.bbrep.2023.101612 (PMC10770592; doi:10.1016/j.bbrep.2023.101612)
Supplement: Multimedia component 1 [file mmc1.docx]

***Supporting information***

Table S1. Limit of detection (LOD) and quantification (LOQ) of the HPLC method. A linear regression analysis was performed by plotting the peak areas of calibrations standards as a function of their calculated concentration (*i.e*., peak area = slope × concentration + y-intercept). The LOD and LOQ were calculated as follows; LOD = 3.3σ/slope and LOQ as LOQ = 10σ/slope, where σ is the standard error of the y-intercept from the regression analysis.

| Cell line | Concentration (μM) | | | |
| --- | --- | --- | --- | --- |
|  | LOD | | LOQ | |
|  | Trp | Kyn | Trp | Kyn |
| HEMa | 0.7 | 0.7 | 2.1 | 2 |
| Mel Juso | 0.7 | 0.6 | 2 | 1.9 |
| Mel Ho | 0.7 | 0.6 | 2 | 1.9 |
| CHL-1 | 1.1 | 0.4 | 3.3 | 1.1 |
| WM-266-4 | 1.1 | 0.4 | 3.3 | 1.1 |
| SK-MEL-3 | 0.8 | 0.4 | 2.3 | 1.3 |
| HT144 | 0.8 | 0.4 | 2.3 | 1.3 |
